# Deep-LC: A Novel Deep Learning Method of Identifying Non-Small Cell Lung Cancer-Related Genes

**DOI:** 10.3389/fonc.2022.949546

**Published:** 2022-07-22

**Authors:** Mo Li, Guang xian Meng, Xiao wei Liu, Tian Ma, Ge Sun, HongMei He

**Affiliations:** Second Affiliated Hospital of Dalian Medical University, Dalian, China

**Keywords:** non-small cell lung cancer, genome-wide association analysis, graph convolutional networks, convolutional neural network (CNN) accelerator, Deep-LC

## Abstract

According to statistics, lung cancer kills 1.8 million people each year and is the main cause of cancer mortality worldwide. Non-small cell lung cancer (NSCLC) accounts for over 85% of all lung cancers. Lung cancer has a strong genetic predisposition, demonstrating that the susceptibility and survival of lung cancer are related to specific genes. Genome-wide association studies (GWASs) and next-generation sequencing have been used to discover genes related to NSCLC. However, many studies ignored the intricate interaction information between gene pairs. In the paper, we proposed a novel deep learning method named Deep-LC for predicting NSCLC-related genes. First, we built a gene interaction network and used graph convolutional networks (GCNs) to extract features of genes and interactions between gene pairs. Then a simple convolutional neural network (CNN) module is used as the decoder to decide whether the gene is related to the disease. Deep-LC is an end-to-end method, and from the evaluation results, we can conclude that Deep-LC performs well in mining potential NSCLC-related genes and performs better than existing state-of-the-art methods.

## Introduction

Statistics show that lung cancer causes 1.8 million deaths each year and remains the leading cause of cancer deaths all over the world (1). Small cell lung cancer (SCLC) and non-SCLC (NSCLC) are two main types. NSCLC accounts for almost 85% of all types of lung cancer (2). Lung cancer has a strong genetic predisposition, and the specific genes are responsible for enhanced risk (3), in addition to being affected by external incentives such as smoking, secondhand or passive smoking, alcohol, and air pollution (4).

Genome-wide association studies (GWASs) have been widely used to identify which genes are related to lung cancer. Hung et al. (5) first utilized GWAS to examine single-nucleotide polymorphisms (SNPs) and discovered a locus in chromosome region 15q25 that was substantially linked to lung cancer. Six genes are found, including three subunits of the nicotinic acetylcholine receptor (CHRNA5, CHRNA3, and CHRNB4). Hu et al. (6) did GWAS on 5,408 subjects and demonstrated that the 5p15 locus is specific to lung cancer. In addition, they found that an independent locus, 22q12.2, may be linked to the susceptibility to lung cancer. Genes are associated not only with the susceptibility to lung cancer but also with lung cancer survival. The 9p21.3 locus was demonstrated to be linked to susceptibility (7) and survival (8).

In addition to GWAS, some studies discovered new variants through next-generation sequencing (NGS), like whole-exome sequencing (WES) and whole-genome sequencing (WGS). Xiong et al. (9) found an uncommon mutation in PARK2 that causes the tumor suppressor gene to lose function in a five-generation family with lung cancer. Exome sequencing of sporadic and familial lung cancer patients also revealed infrequent detrimental mutations in GWAS-nominated sites in DBH and CDC147 genes (10).In a family with a high prevalence of lung adenocarcinoma, it was found that a functional missense mutation in the oncogene YAP1 was linked to the likelihood of getting the illness through WGS (11).

With a more comprehensive understanding of genes, more and more studies take gene interaction information into account. Maurano et al. (12) demonstrated that the regulation relationship between genes plays a vital role in the disease research field. Although GWAS, WES, and WGS demonstrated the effectiveness of mining disease-related genes in previous studies, this method ignores a large amount of complex information about interactions between gene pairs. Interaction networks have proven effective in the field of biological information, like identifying disease-related molecules (13) and predicting protein–metabolite interactions (14). Graph convolutional network (GCN) (15) is one type of neural network architecture to learn nodes and edges of graphs. It has been proved that GCN enhances algorithms of abilities to mine information and make decisions in the bioinformatics field. For example, Deep-DRM was proposed to identify disease-related metabolites (16). In Deep-DRM, GCN was applied as an encoder to integrate features of metabolites and disease. In DeepLGP, GCN was applied to convolve a gene interaction network for encoding the features of genes and lncRNAs (17). Cheng et al. (18) proposed a deep learning method to predict cell type-specific genes of lung cancer based on SC2disease (19) and other databases. This task only inferred cell type-specific genes of lung cancer in 8 cell types, instead of directly demonstrating whether the gene is related to lung cancer.

Interaction relationships of genes can be translated into a graph network. We treated the task of identifying NSCLC-related genes as a binary classification and proposed a novel deep learning method named DEEP-LC to solve it. GCN was applied to learn and extract relevant features from gene interaction networks, and CNN was the classification module to identify target genes.

## Method

**Figure 1:**
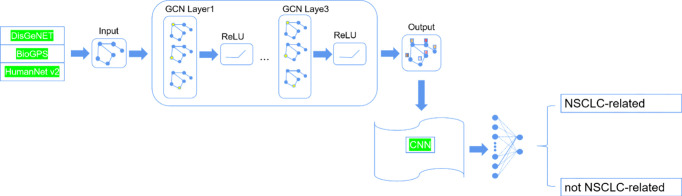
The structure of Deep-LC.

The method called Deep-LC that we proposed includes two parts. The structure is shown in **Figure 1**. First, we constructed a graph network by gene interaction information related to lung cancer and used a GCN to extract features of interaction information between genes. Then, we constructed a small convolutional neural network (CNN) to identify potential lung cancer-related genes.

### Construction of the Graph Network of Genes

The graph network of genes represents the genes interaction network. The graph network contains nodes and edges. In the study, the genes that we selected are the nodes, and interaction information between gene pairs is the edges. Interactions information was obtained from the public database. It should be noted that outliers that had no interaction with other genes were removed.

### Extracting Features by Graph Convolutional Network

Since the interactions between genes were expressed by the gene network, we use a GCN to extract features from the gene network. The graph network we built can be expressed as G = (V,E,W). V represents the nodes of the network, E represents the edges, and W represents the weighted matrix encoding the connection weight between vertices.

The Laplacian matrix is defined as


(1) L=D−A

where D means the degree matrix of the network and A is the adjacency matrix.

Since the features of genes should contain not only connections between nodes but also the information itself, we can get


(2) $$A^{\prime }=A+I$$

where *I* is the identity matrix.

Then the inverse degree matrix *D*’ can be obtained.


(3) $$D^{\prime }=\sum A^{\prime }$$

Last, we can get the features, as follows:


(4) $$X^{\prime }=\sigma \left({D^{\prime }}^{\frac{1}{2}}A^{\prime }{D^{\prime }}^{\frac{-1}{2}}X\right)$$

where *X* is the features map of each node and *σ* is the activation function. In the study, we use rectified linear unit (ReLU) function as the activation function. The expression is as follows:


(5) ReLU=max(x, 0)

### Identifying Non-Small Cell Lung Cancer-Related Genes by Convolutional Neural Network

CNN excels at computer vision and is gaining traction in the field of bioinformatics. In comparison to a pure deep neural network, CNN performs better due to the following characteristics: 1) by utilizing the sparsity of connections and parameter sharing, the convolutional layer has fewer parameters. In other words, under the same amount of parameters, CNN is superior at mining and learning characteristics from nodes. 2) The convolutional layer gathers data from both global and local features. Because the features of disease-related genes focused on some specific areas, global features are redundant when it comes to identifying disease-related genes. As a result, studying local features can assist us in extracting crucial information from features. Therefore, CNN is applied as the supervised model to decide which genes are associated with NSCLC in the study.

The structure of the CNN is shown in **Table 1**. Our CNN module has four convolutional layers and a full-connected layer. We still used ReLU as the activation function the same as the GCN. Between layers, we added batch normalization (20) to avoid gradient disappearance and gradient explosion and avoid over-fitting. Both the above layers strengthen the ability of the features fusion learning and decision making.

**Table 1:** The structure of CNN.

Layers	Kernel size	The number of filters
Convolutional layer	3	32
Batch normalization/ReLU		
Convolutional layer	3	64
Batch normalization/ReLU		
Convolutional layer	3	32
Batch normalization/ReLU		
Convolutional layer	3	16
Batch normalization/ReLU		

CNN, convolutional neural network; ReLU, rectified linear unit.

It should be noted that the activation function we used after the full-connected layer is softmax function. Because our task is the binary classification task, we used binary cross-entropy as the loss function, as follows:


(6) $$\text{Loss}=-y_{i}log\left(p_{i}\right)-\left(1-y_{i}\right)log\left(1-p_{i}\right)$$

where *y_i_* means the true value and *p_i_* means the predicted value.

For training details, we used dropout to avoid over-fitting, and we set the rate at 0.5. We used Adam with default parameters as the optimization algorithm. We trained our method 50 epochs. The initial learning rate is 0.01 and reduced to 1/10 after 40 epochs.

## Result

### Datasets

In the paper, we selected genes that are related to NSCLC disease from DisGeNET (21), which is a platform that integrates information on gene–disease associations. NSCLC includes stage I, II, III, IIIA, and IIIB types; they are 115, 11, 16, 12, and 11 disease-related genes for the different types of NSCLC, respectively. After integrating the same genes with different types of NSCLC, we obtained 142 NSCLC-related genes. We obtained gene expression of different tissues from BioGPS (22). After deleting genes that lacked information on the probe set, we obtained 142 positive samples finally. Considering data balance, we randomly selected 142 genes that were reported as not being related to NSCLC as the negative samples. Then we obtained interactions between genes from the HumanNet database (23). In the gene interaction network, the nodes are genes that we selected, and the edges are interactions between gene pairs. In the paper, we used log likelihood score (LLS) as the weight of the edges because these scores can represent the interactions between genes.

### Experiment Setup

Cross validation was used to demonstrate the performance of the algorithm in the study. The fold number was set to 10. Specifically, the dataset including the test set and the train set was divided into 10 subsets. One subset was randomly selected as the test set, and the remaining subsets were selected as the train set. In other words, every experiment was repeated 10 times totally in the paper.

The task of identifying lung cancer-related genes can be treated as a binary classification problem. The precision–recall curve is plotted based on different precision and recall, and the receiver operating characteristic curve (ROC curve) is based on different recall and false-positive rates (FPRs). Precision, recall, and FPR can be calculated as follows:


(7) $$\text{Precision}=\frac{TP}{TP+FP}$$

(8) $$\text{Recall}=\frac{TP}{TP+FN}$$

(9) $$\text{FPR}=\frac{FP}{FP+TN}$$

where *TP* is a true positive, *FP* is a false positive, *FN* is a false negative, and *TN* is a true negative. We used the area under the precision–recall curve (AUPR) and the area under the ROC curve (AUC) as evaluating indicators. AUPR and AUC can help us demonstrate the effectiveness of the classification algorithm.

### Performance

Stacking N-level GCN layers can convolve information from its N-order neighbors. Stacking too many GCN layers may lead to the vanishing gradient problem (24). Too little layers may cause feature learning insufficiency. So we evaluated the influence of different numbers of GCN layers on Deep-LC. The results are shown in **Table 2**. Deep-LC with three GCN layers has the best performance.

**Table 2:** The performance of Deep-LC with different of GCN layers.

Layers	AUC	AUPR
1	0.7051	0.7264
2	0.7895	0.7708
3	0.8017	0.7893
4	0.7643	0.7329

GCN, graph convolutional network; AUC, area under the receiver operating characteristic curve; AUPR, area under the precision–recall curve.

If the number of layers is more than three, both AUC and AUPR scores decrease. This result might be related to the gradient vanishing problem to some extent. We can conclude that the performance of the Deep-LC method is enhanced by stacking layers. This operation can strengthen the capability of feature fusion and be helpful for feature mining.

### Comparison Experiments

**Figure 2:**
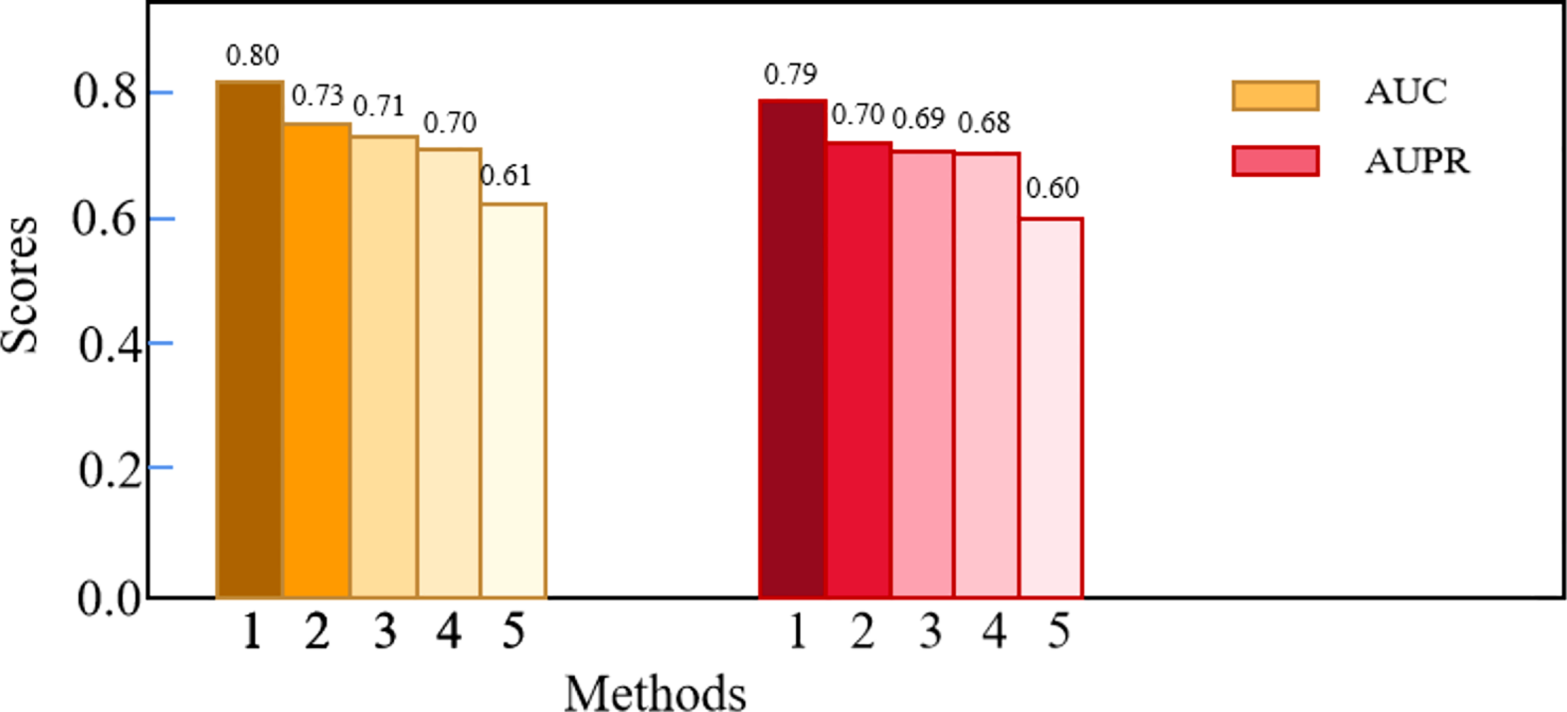
The comparison results of Deep-LC and other four methods.

We compared Deep-LC with the other four methods, including GCN, CNN, random forest (RF), and K-nearest neighbor (KNN). **Table 3** shows the specific results, and **Figure 2** depicts the outcomes.

**Table 3:** The AUC and AUPR scores of Deep-LC and other four methods.

Method	AUC	AUPR
Deep-LC	0.8017	0.7893
GCN	0.7343	0.7028
CNN	0.7122	0.6855
RF	0.6965	0.6834
KNN	0.6137	0.5962

AUC, area under the receiver operating characteristic curve; AUPR, area under the precision–recall curve; GCN, graph convolutional network; CNN, convolutional neural network; RF, random forest; KNN, K-nearest neighbor.

According to the results of the trial, Deep-LC outperforms all other approaches in terms of AUC and AUPR scores of 0.8017 and 0.7893. As compared to GCN, CNN, RF, and KNN, Deep-LC’s AUC scores increase by 9.18%, 12.56%, 15.09%, and 30.63%, respectively, and AUPR scores rise by 12.31%, 15.13%, 15.49%, and 32.38%, respectively. KNN had the lowest results, with AUC and AUPR of 0.6137 and 0.5962, respectively. In conclusion, the results reveal that Deep-LC outperforms various state-of-the-art approaches in terms of identifying NSCLC-related genes. The performance of using GCN and CNN is better than using one alone.

## Case Study

To further demonstrate the effectiveness of Deep-LC, we did case studies. We aimed to identify some genes that may be related to NSCLC disease and not a positive sample that we selected. At last, we found several genes and relevant papers to support them. **Table 4** lists the genes.

**Table 4:** The details of genes that we mined by Deep-LC method.

Name	Entrez ID	References
KLK10	5655	Zhang et al. proved that KL10 was considerably downregulated in NSCLC compared to non-cancer samples. They concluded that KLK10 functions as a tumor suppressor gene in NSCLC, and epigenetic inactivation is a common occurrence in NSCLC pathogenesis that could be exploited as a biomarker (25).
DLEC1	9940	The study found that expression levels of DLEC1 were significantly different between tumor and normal tissues (p = 0.0001) (26).
EFEMP1	2202	EFEMP1 found a significantly higher frequency of methylation in NSCLC compared with the normal tissues (p ≤ 0.001) (27).

NSCLC, non-small cell lung cancer.

## Conclusion

Lung cancer is the main cause of cancer mortality worldwide. NSCLC accounts for over 85% of all lung cancers. GWAS and NGS have been used to discover genes related to NSCLC. However, many studies ignored the intricate interaction information between gene pairs. In the paper, we proposed a novel deep learning method named Deep-LC for identifying NSCLC-related genes. We treated the task as a binary classification problem and integrated information to build a gene interaction network. GCNs were applied as an encoder to extract features of gene interactions network, and a simple CNN module was applied as the decoder to decide whether the gene is related to the disease. Deep-LC is an end-to-end method, and from the evaluation results, we can conclude that Deep-LC performs better than existing state-of-the-art methods.

## Data Availability Statement

The original contributions presented in the study are included in the article/supplementary material. Further inquiries can be directed to the corresponding authors.

## Author Contributions

ML and GM designed the experiments, analyzed the data, and wrote the manuscript. XL and TM analyzed the bioinformatic data. GS provided important ideas. This whole work is guided by HH. All authors contributed to the article and approved the submitted version.

## Funding

This study was supported by the Science and Technology funds from Liaoning Education Department (No. LZ2020038 ) and Wu Jieping Medical Foundation (No. 320.6750.2021-16-47).

## Conflict of Interest

The authors declare that the research was conducted in the absence of any commercial or financial relationships that could be construed as a potential conflict of interest.

## Publisher’s Note

All claims expressed in this article are solely those of the authors and do not necessarily represent those of their affiliated organizations, or those of the publisher, the editors and the reviewers. Any product that may be evaluated in this article, or claim that may be made by its manufacturer, is not guaranteed or endorsed by the publisher.
